# Experimental and Numerical Study on the Perforation Behavior of an Aluminum 6061-T6 Cylindrical Shell

**DOI:** 10.3390/ma16217055

**Published:** 2023-11-06

**Authors:** Seon-Woo Byun, Young-Jung Joo, Soo-Yong Lee, Sang-Woo Kim

**Affiliations:** 1 Advanced Technology R&D Center, Design Analysis Team, Hanwha Aerospace, 6, Pangyo-ro 319beon-gil, Bundang-gu, Seongnam-si 13488, Gyeonggi-do, Republic of Korea; sunwoo1053@naver.com (S.-W.B.); tj07000@gmail.com (Y.-J.J.); 2 School of Aerospace and Mechanical Engineering, Korea Aerospace University, 76 Hanggongdaehak-ro, Goyang-si 10540, Gyeonggi-do, Republic of Korea; leesy@kau.ac.kr; 3 Research Institute for Aerospace Engineering and Technology, Korea Aerospace University, Goyang-si 10540, Gyeonggi-do, Republic of Korea

**Keywords:** high-velocity impact, modified Johnson–Cook model, impact energy, strain rate

## Abstract

The modified Johnson–Cook (MJC) material model is widely used in simulation under high-velocity impact. There was a need to estimate a strain rate parameter for the application to the impact analysis, where the method typically used is the Split Hopkinson bar. However, this method had a limit to the experiment of strain rate. This study proposed to estimate the strain rate parameter of the MJC model based on the impact energy and obtained a parameter. The proposed method of strain rate parameter calculation uses strain parameters to estimate from the drop weight impact and high-velocity impact experiments. Then, the ballistic experiment and analysis were carried out with the target of the plate and cylindrical shape. These analysis results were then compared with those obtained from the experiment. The penetration velocities of plates could be predicted with an error of a maximum of approximately 3.7%. The penetration shape of the cylindrical target has a similar result shape according to impact velocity and had an error of approximately 6%.

## 1. Introduction

Safety evaluations required by the Federal Aviation Administration (FAA) and the European Aviation Safety Agency (EASA) for the operation of a developed aircraft engine include an evaluation of the containment of aircraft engines. The blades of aircraft engines have a strong rotational force. For this reason, if a damaged blade inside the engine penetrates the engine case, aircraft safety is a major threat. The objective of the containment case of an aircraft engine is to prevent blade debris from being flung outward. Therefore, it is essential to evaluate the containment of the engine case to protect the aircraft from damaged blade fragments [1,2]. 

The experimental methods for containment evaluation consist of high-velocity rotation and ballistic impact experiments. A high-velocity rotation experiment simulates the actual rotation of an aircraft engine blade. The blade has a notch that breaks at a specific speed and causes damage when the rotor is spun. Le [3] evaluated the containment of an aircraft engine using a high-velocity rotation experimental device. He et al. [4] evaluated the containment using a high-velocity rotation experiment of a soft-wall aircraft engine made of composite and metallic materials. Although this study could accurately identify the damage to the blade fragment and the inside of the engine case, it was difficult to predict the speed at which blade damage occurred [5,6].

The ballistic impact experiment is an evaluation method for the containment and penetration of blade fragments by generating an impact using a gas gun. Naik et al. [7] used a ballistic experiment to determine the safety of a containment case made of fabric composite to protect against penetration. Carney et al. [8] proposed a method for conducting blade ballistic impact experiments on plates with convex curved geometries. This experimental method has the advantage of a simple setup for the projectile speed and adjustment of the collision angle; however, it is difficult to evaluate the behavior of an actual broken blade [9,10]. In addition, the experimental method for evaluating the containment of this engine is performed before the final experiment using a reduced or partial model of the developed engine. 

The analytical method evaluates the containment case through an impact analysis using a finite element method (FEM). The Johnson–Cook (JC) material model has been widely used to model material behavior subjected to high-velocity impacts [11]. The JC material model is commonly used to simulate the high-velocity impact behavior in the analysis of blade containment; the coefficients of this model were obtained using static and dynamic experiments. Yuriy et al. [12] used the ANSYS program to analyze the behavior of a damage to the aircraft engine case. Since the Johnson-Cook model commonly used to simulate high-velocity impact behavior in the analysis of blade containment offer of aircraft engines, the coefficients of this model were obtained using static and dynamic experiments. Lee et al. [13] determined the Split Hopkinson bar (SHPB) of an aluminum casing using the parameters of an aluminum alloy from the JC material model. Ambur et al. [14] verified the JC material model coefficients of titanium 6AI-4V and aluminum 2024 after comparing them with the results of an impact experiment. This study confirmed the shape of the damage in the case of impact. A verification process using experiments is required [15,16]. The JC material model assumes linear strain rate-stress relationships, leading to inaccurate results when compared with high-velocity impact experiments due to the linear increase in flow stress with strain rate. Huh and Kang [17] verified through their experiments that this linear relationship inadequately describes material behavior at high strain rates. To address this issue, the modified Johnson–Cook (MJC) material model was introduced, assuming a non-linear strain rate-stress relationship. Choudhary et al. [18] performed penetration analysis using the MJC material model. It was confirmed that an accurate penetration shape was predicted. The coefficient of the MJC material model is obtained by quasi-static and dynamic experiments. Also, the strain coefficient is typically calculated by dynamic experiments using SHPB. However, this experimental method has a limitation of the strain rate (10^4^ s^−1^), and the accuracy of the strain rate coefficient depends on the number of experiment results [19,20]. Therefore, Wang et al. [21] calculated the strain rate coefficient of the optimal JC material model by analyzing the finite element based on the fine geometry through ball impact experiments. Burley et al. [22] estimated the strain rate coefficient of the JC material model by comparing it with the results of finite element analysis based on the fine depth and shape by impact experiments. Also, the analysis research on containment systems requires accurate penetration prediction of blades that are conducted over a long time and impact at high velocity.

In this study, the modified Johnson–Cook (MJC) material and Cockcroft–Lathan (CL) fracture models were used for ballistic impact analysis. Furthermore, a strain rate coefficient was determined using an impact energy-based estimation method to enhance the accuracy of penetration velocity prediction. This is particularly important because high-velocity impact loads exhibit distinct characteristics compared to material behavior under static loads, with a significant influence from the elevated strain rates. The coefficients of the MJC material and CL fracture models were obtained via experiments using the universal testing machine, drop-weight impact equipment, and high-velocity impact equipment. A ballistic impact analysis was conducted using the LS-DYNA, R12.0.0. The ballistic impact experiment used high-velocity impact equipment of the gas gun type. The model coefficient was verified by comparing the experimental results and analysis results. And the damage shape on the cylindrical shell is predicted as a result. The damage shape and penetration velocity of the projectile in the experiment were compared with the analysis results. By using this approach, the containment evaluation of an aircraft engine case is verified by cross-checking with the analytical and experimental results.

## 2. Material Models

### 2.1. Cockcroft–Lathan Fracture Model

**Figure 1:**
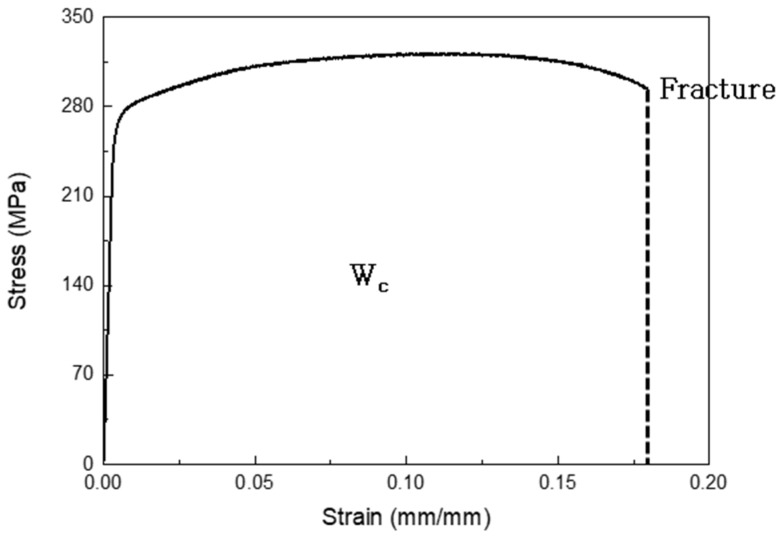
Coefficient of Cockcroft–Lathan fracture model by quasi-static experiment.

The CL fracture model was a strain energy-based fracture model proposed by Cockcroft and Lathan [23]. The analytical method of high-velocity impact requires a material damage simulation, for which the CL fracture model is applied. It can be expressed as the strain energy in the form (Equation (1)). Also, this model is assumed to have the same strain energy according to the strain rate. Element failure is started from the damage index of 1. This means that the fracture occurs at the same strain energy, even at a high strain rate, based on the strain energy obtained in the quasi-static experiment. Thus, it had the advantage that the coefficient value was small compared to the other fracture models.

(1) $$D=\frac{W}{W_{C}}=\frac{1}{W_{C}}\int_{0}^{\varepsilon_{f}}\sigma d\varepsilon$$

where *D* is the damage index, *W_C_* is the reference strain energy, $\varepsilon_{f}$ is the fracture strain, and *σ* is the tensile stress. The reference strain energy was calculated in the quasi-static experiment, which employed the MTS-810 at a strain rate of 0.01 s^−1^ (Figure 1).

### 2.2. Modified Johnson–Cook Material Model

**Figure 2:**
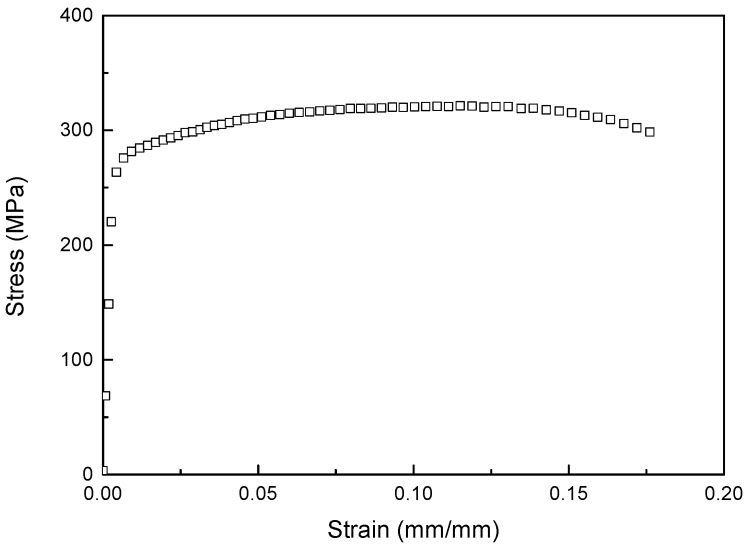
Experimental results showing the strain hardening.

The MJC material model is expressed as a simple product of the strain hardening, the strain rate, and the thermal softening terms (Equations (2) and (3)). The coefficients of the MJC material model are obtained by quasi-static and dynamic experiments [24].

(2) $$\sigma_{Y}=[A+B\bar{\varepsilon_{p}^{n}}]{\left[1+{\dot{\varepsilon }}^{*}\right]}^{C}[1-{\left(T^{*}\right)}^{m}]=\sigma_{H}{\left[1+{\dot{\varepsilon }}^{*}\right]}^{C}[1-{\left(T^{*}\right)}^{m}]$$

(3) $$\begin{array}{cc} {\dot{\varepsilon }}^{*}=\frac{\bar{\varepsilon_{p}}}{{\dot{\varepsilon }}_{0}}, & T^{*}=\frac{T-T_{r}}{T_{m}-T_{r}} \end{array}$$

where *σ_Y_* is the dynamic yield stress, *A* is the initial yield stress, and *B* and *n* are the strain hardening parameters. $\bar{\varepsilon_{p}^{n}}$ is the equivalent plasticity strain, *C* is the strain rate sensitivity parameters, ${\dot{\varepsilon }}^{*}$ is the strain rate, T* is the equivalent temperature, *m* is the thermal softening index, and *σ_H_* is the strain hardening term. However, the effect of temperature is not considered because of room temperature. The coefficients for the strain hardening terms (*A*, *B*, and *n*) are derived from quasi-static experiments, which depict material behavior following plastic stress. The quasi-static experiment measured the plastic behavior that is the reference for flow stress. This study used an MTS-810 at a strain rate of 0.01 s^−1^. The specimen was tested at a speed of 30 mm/min, as specified by the reference gauge length. The strain was measured with an extensometer (Model 3542). Therefore, the coefficients of strain hardening were obtained by fitting the data after the yield stress. The experimental process followed the guidelines of ISO 26203-2 [25]. The coefficients of the strain hardening term (*A*, *B*, and *n*) were obtained from the experimental results (Figure 2).

The coefficients for the strain rate term (*C*) are determined through dynamic experiments, typically utilizing the SHPB method, where strain rates range from 500 to 3000 s^−1^. Strain and stress are measured using strain gauges on the input bar, output bar, and specimen. These experiments reveal that the influence of strain rate on material plastic stress becomes more pronounced as the strain rate increases. However, for high-velocity impact analysis, a more comprehensive understanding of material behavior at even higher strain rates is required. The coefficient of strain rate term used an impact energy-based equation by ball impact experiment for high strain rate. It needs low-velocity impact and high-velocity impact experiments for this method [26]. The impact energy equation was composed of initial impact, rebound, strain, and deflection energy (Equation (4)).

(4) $$U_{\mathrm{Imp}}-U_{\mathrm{Reb}}-U_{\mathrm{Str}}-U_{\mathrm{Def}}=0$$

where *U*_Imp_ is the initial impact energy, *U*_Reb_ is the rebound energy, *U*_Str_ is the strain energy, and *U*_Def_ is the deflection energy. 

The initial impact energy is calculated as the mass and initial impact velocity of the projectile. Equation (5) is the initial impact energy equation. The rebound energy is estimated as the rebound velocity of the projectile (Equation (6)). The deflection energy is calculated as the deflection displacement and force (Equation (7)).

(5) $$U_{\mathrm{Imp}}=\frac{1}{2}mv^{2}$$

(6) $$U_{\mathrm{Reb}}=\frac{1}{2}mv^{\prime 2}$$

(7) $$U_{\mathrm{Def}}=\int p_{d}(t){\mathrm{du}}_{0}$$

where *m* is the mass of the projectile, *v* is the initial impact velocity, *v’* is the rebound velocity, *p_d_*(*t*) is the deflection force, and *u*_0_ is the deflection displacement. 

**Figure 3:**
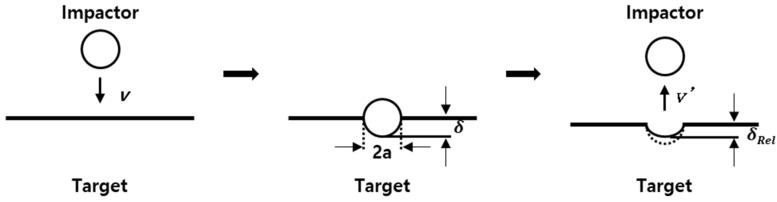
Schematic diagram of the ball impact process.

The strain energy was the energy by plastic strain. It was calculated by the strain and impact pressure in the process of applying the impact load. Equations (8)–(10) are expressed in the contact pressure and penetration volume [27].

(8) $$U_{\mathrm{str}}=\int_{0}^{\delta^{*}}p\pi a^{2}d\delta =pV$$

(9) $$V=\pi (R\delta^{2}-\frac{1}{3}\delta^{3})$$

(10) $$p=3\sigma_{y}+\frac{3}{10}\rho_{t}{\bar{v}}^{2}$$

where *p* is the dynamic contact pressure, *V* is the penetration volume, *R* is the radius of the projectile, *δ* is the indentation depth, *σ_y_* is the dynamic yield stress, *ρ_t_* is the density of the target, and $\bar{v}$ is the mean impact velocity. Equations (11) and (12) convey the dynamic yield stress in terms of energy, while Figure 3 visualizes the impact of the ball.

(11) $$U_{\mathrm{Imp}}-U_{\mathrm{Reb}}-U_{\mathrm{Def}}-U_{Str}=U_{\mathrm{Imp}}-U_{\mathrm{Reb}}-U_{\mathrm{Def}}-(3\sigma_{y}+\frac{3}{10}\rho_{t}{\bar{v}}^{2})V=0$$

(12) $$\sigma_{y}=\frac{U_{\mathrm{Imp}}-U_{\mathrm{Reb}}-U_{\mathrm{Def}}}{3V}+\frac{3}{10}\rho_{t}{\bar{v}}^{2}=\sigma_{H}{\left[1+{\dot{\varepsilon }}^{*}\right]}^{C}$$

The ball impact experiments were used to determine the coefficient of strain rate term. The mass of the low-velocity impact experimental projectile was heavier than that of the high-velocity impact experimental projectile, and the impact experiment was setting the same impact energy for considering only the strain rate effect. The dynamic yield stress for low-velocity impacts initially disregarded the strain rate effect and assumed quasi-static behavior (as indicated in Equation (13)). This assumption was made despite the presence of real dynamic characteristics in low-velocity impact experiments. Nevertheless, it is important to note that the calculated strain rate in these experiments is around 10^2^ s^−1^, which places it at the interface between quasi-static and dynamic loading conditions. The impact of dynamic characteristics is relatively minor at this boundary, thus supporting the decision to treat it as quasi-static behavior. The dynamic yield stress of high-velocity impact was affected by the strain rate (Equation (14)).

(13) $$\sigma_{y1}=\frac{U_{\mathrm{Imp}1}-U_{\mathrm{Reb}1}-U_{\mathrm{Def}1}}{3V_{1}}=\sigma_{H1}$$

(14) $$\sigma_{y2}=\frac{U_{\mathrm{Imp}2}-U_{\mathrm{Reb}2}-U_{\mathrm{Def}2}}{3V_{2}}+\frac{3}{10}\rho_{t}{\bar{v}}^{2}=\sigma_{H2}{\left[1+{\dot{\varepsilon }}^{*}\right]}^{C}$$

The plastic strain was determined based on the indentation size. In this scenario, the actual indentation size created by the high-velocity impact experiment’s projectile was assumed to be similar to that produced by the low-velocity impact experiment’s projectile due to their identical impact energy. Nevertheless, it is crucial to recognize that the strain rate was contingent on the velocity at which the indentation was formed, and this rate varied depending on the strain rate conditions. Equations (15) and (16) are the strain rate relation. The strain hardening term was the same for the low-velocity and high-velocity impacts because strain hardening and strain rate terms of the MJC material model are independent.

(15) $${\dot{\varepsilon }}^{*}=\frac{{\dot{\varepsilon }}_{p}}{{\dot{\varepsilon }}_{0}}=\frac{{\dot{\varepsilon }}_{p2}}{{\dot{\varepsilon }}_{p1}}=\frac{\frac{d\varepsilon_{p2}}{d\delta }v_{2}}{\frac{d\varepsilon_{p1}}{d\delta }v_{1}}\approx \frac{v_{2}}{v_{1}}$$

(16) $${\dot{\varepsilon }}_{p}=\frac{d\varepsilon_{p}}{dt}=\frac{d\varepsilon_{p}}{d\delta }\frac{d\delta }{dt}=\frac{d\varepsilon_{p}}{d\delta }v$$

Equation (17) is estimating for the coefficient of strain rate term (MJC model).

(17) $${\left[1+{\dot{\varepsilon }}^{*}\right]}^{C}={\left[1+\frac{v_{1}}{v_{2}}\right]}^{C}=\frac{\sigma_{y2}}{\sigma_{H1}}$$

where subscript 1 is expressed as the low-velocity impact and subscript 2 is expressed as the high-velocity impact. To summarize, in the context of low-velocity impact, the strain hardening term aligns with the flow stress. This flow stress is calculated using energy and indentation volume. In the case of high-velocity impact, the flow stress is derived from a combination of factors, including energy, indentation volume, target density, and impact velocity. Furthermore, the calculation of the strain rate coefficient in the MJC model was also performed. 

### 2.3. Validation of Material Models

**Figure 4:**
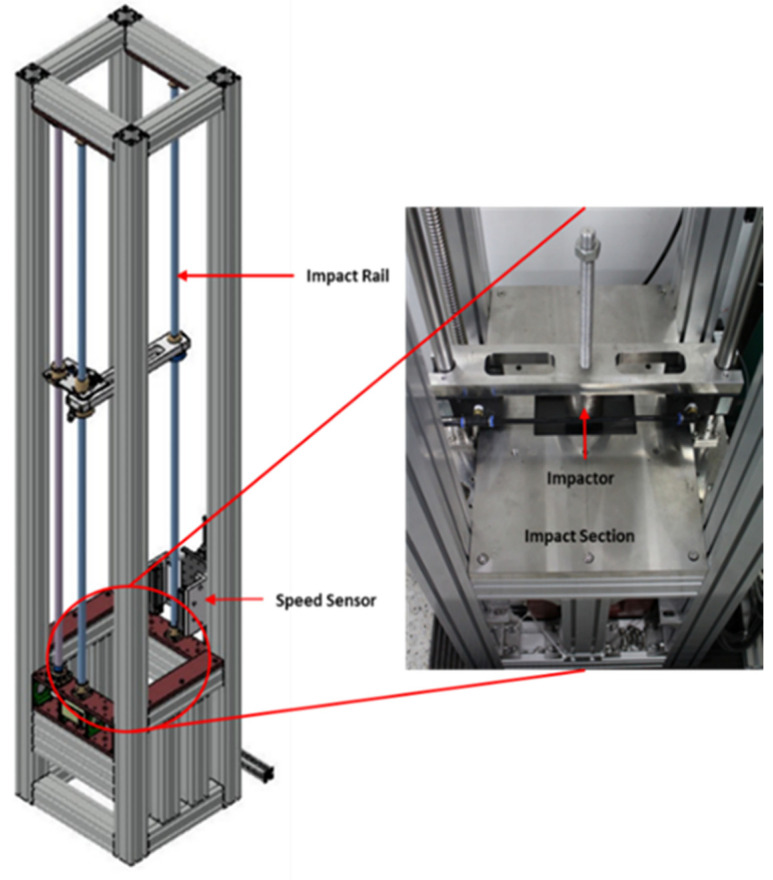
Drop weight impact equipment for low-velocity impact experiment.

**Figure 5:**
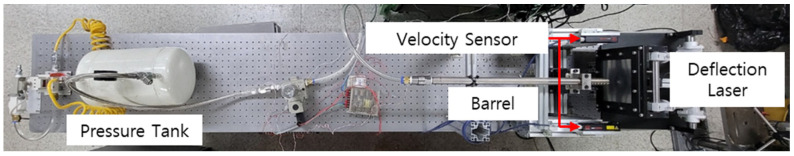
High-velocity impact experimental equipment.

**Figure 6:**
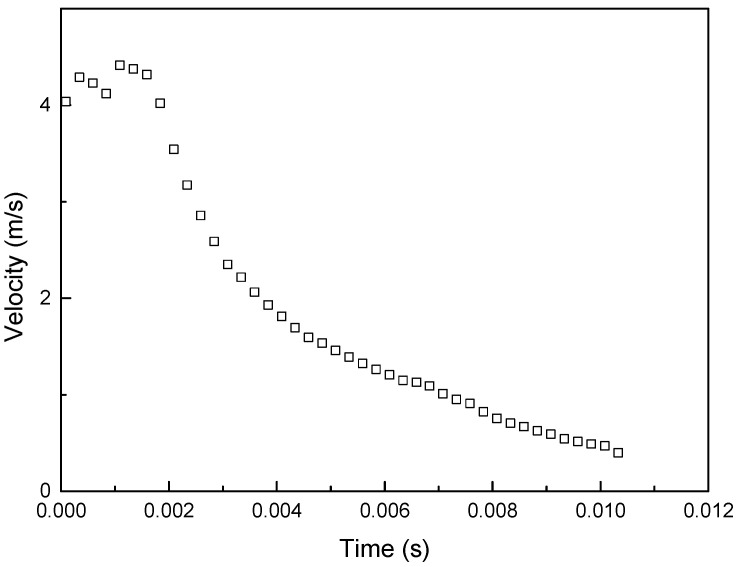
Experimental result based on the low-velocity impact (v = 4.2 m/s).

**Figure 7:**
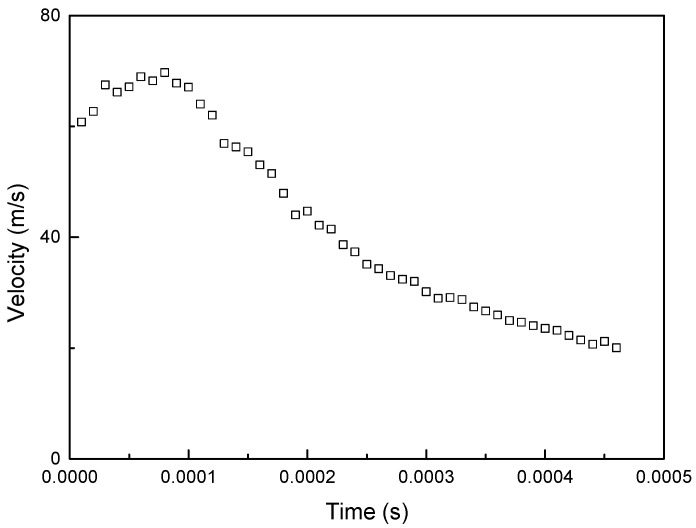
Experimental result based on the low-velocity impact (v = 71.4 m/s).

To validate the material models, we conducted a ball impact experiment, as shown in Figure 4 and Figure 5. This experiment involved low-velocity impact and employed drop-weight impact equipment. The impact velocity was measured by the velocity sensor (ODC 1200/90), and the deflection displacement was measured by the displacement sensor of the laser type. The impact velocity was measured by the velocity sensor (ODC 1200/90), and the deflection displacement was measured by the displacement sensor of the laser type. The low-velocity impact experiments were conducted at a speed lower than 10 m/s. The high-velocity impact experiments were conducted at speeds ranging from 50 m/s to 1000 m/s. The impact velocity was measured at 4.2 and 71 m/s, as shown in Figure 6 and Figure 7. The mass of the projectile was 3 kg in the low-velocity impact experiment, and the mass of the projectile was 0.012 kg in the high-velocity impact experiment. The specimen of low-velocity impact measured 0.15 m × 0.1 m, and thickness was 0.003 m. The specimen of high-velocity impact measured 0.19 m × 0.9 m, and thickness was 0.003 m. Table 1 lists the impact velocity. This strain rate was measured at 500 s^−1^ and 12,000 s^−1^ after each experiment. As a result, the coefficients of the MJC material and the CL fracture models could be obtained and are presented in Table 2. Table 3 lists the material properties of the aluminum 6061-T6.

**Figure 8:**
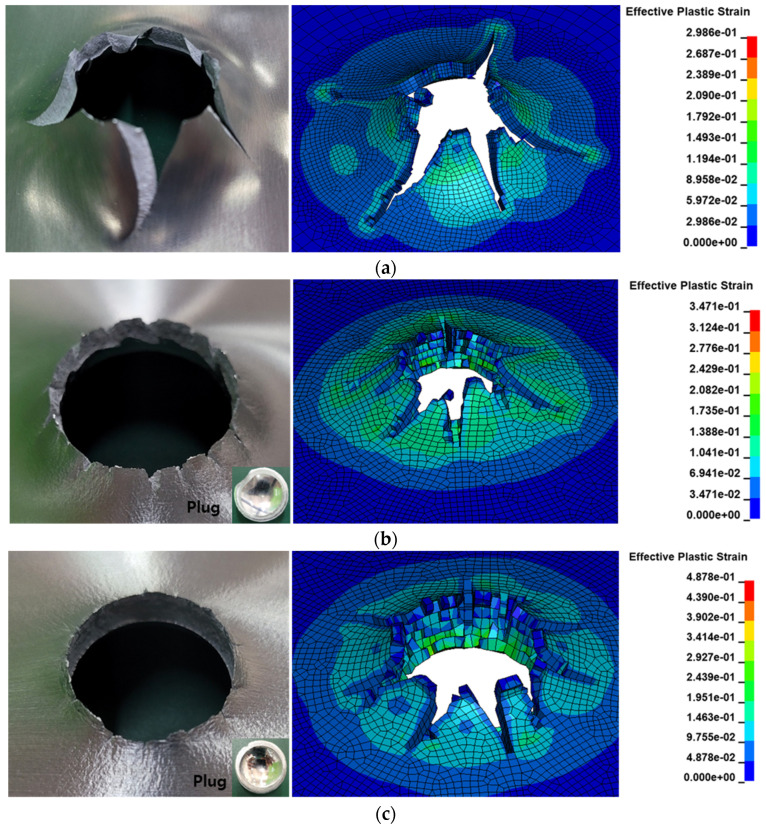
Damaged shapes of experiment and analysis results: (**a**) thickness: 3 × 10^−3^ m; (**b**) thickness: 6 × 10^−3^ m; (**c**) thickness: 8 × 10^−3^ m.

**Figure 9:**
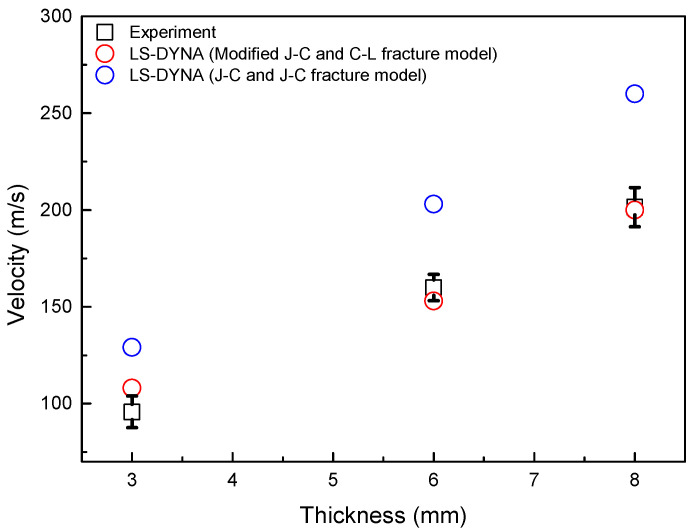
Penetration velocity based on the thickness of the plate according to analysis models.

The coefficient verification of the MJC material and CL fracture model was performed by comparing the results of the experiment and the analysis on the aluminum 6061-T6 plate. The plate measured 300 mm × 300 mm. The thicknesses of the plates were 3 mm, 6 mm, and 8 mm. The projectile was a steel ball (diameter: 25 mm) of 64 g. The ballistic impact analysis results revealed the critical velocity at which penetration occurred, which was compared with the results of the experiments (penetration/nonpenetration velocity) to verify the coefficient, as illustrated in Table 4 and Figure 8. The Cl fracture model simulated excessive damage. It seems that the Cl fracture model is caused by not implementing the spalling fracture mode. Figure 9 illustrates a comparative analysis between the JC material and JC fracture models and the MJC material and CL fracture models alongside experimental results. The critical velocity at which the predicted penetration occurs by applying the coefficients of the MJC material and CL fracture model corresponds to the nonpenetration/penetration velocity range of the ballistic impact experiment. 

**Figure 10:**
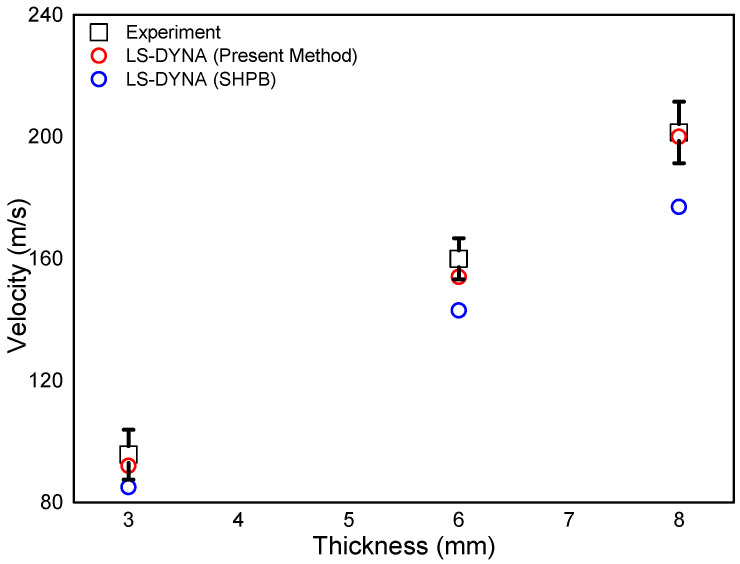
Penetration velocity based on the thickness of the plate according to method.

The strain rate coefficient, obtained through both the SHPB and our proposed method, underwent validation. Figure 10 illustrates that our method provides a more accurate prediction of penetration velocity, confirming the effectiveness of the strain rate coefficient obtained through the combined experimental and computational approach presented in this study for accurate penetration velocity prediction.

## 3. Ballistic Impact Experiments

**Figure 11:**
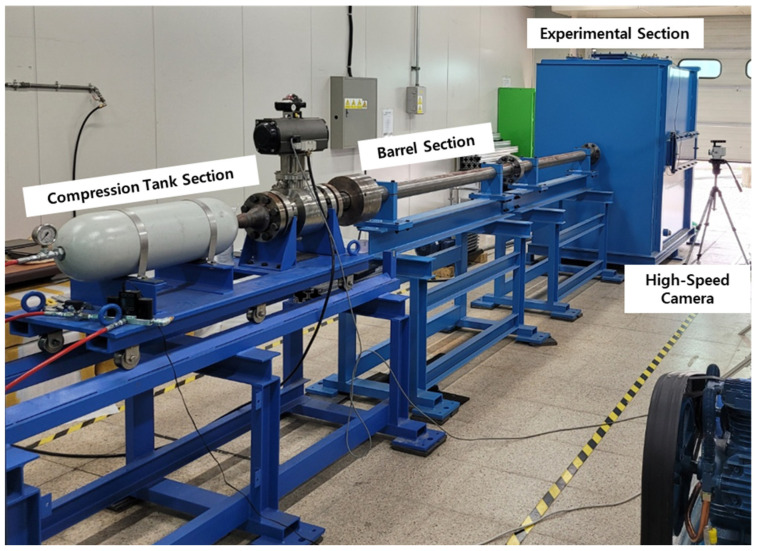
Ballistic experiment equipment.

**Figure 12:**
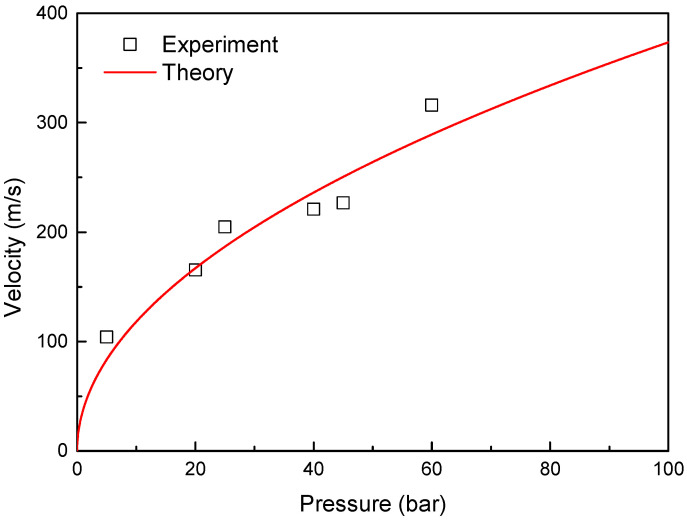
Comparison of calculated velocity and experimental velocity.

The equipment used in the ballistic impact experiment included compression tanks, barrels, and experimental sections, as depicted in Figure 11. The projectile was launched by applying a pressure of up to 100 bar. The experimental equipment featured a 4 m barrel with a diameter of 0.08 m. By using a 150 g projectile filled with helium gas at 70 bar pressure, we achieved a maximum velocity of approximately 380 m/s. Velocity was measured using a high-velocity camera (Phantom VEO E310L-18G-C). The impact images were recorded at 20,000 frames per second. The experimental environment was created with a vacuum of −0.96 bar to prevent resistance and shock. The injection velocity of the projectile under gas pressure can be predicted using Equation (18) [15].

(18) $$v_{2}=\sqrt{\frac{2P_{1}V_{1}}{m_{p}R}(1-{\left(\frac{V_{1}}{V_{2}}\right)}^{n-1})(c_{v}+\frac{R}{1-n})}$$

where *R* is the gas constant, *C_v_* is the specific heat at constant volume, *n* is the polytropic index, *V*_1_ is the volume of the pressure tank, *V*_2_ is the volume after injection, *P*_1_ is the pressure of the tank, and *v*_2_ is the injection velocity. Figure 12 shows a comparison of theoretical and experimental velocities. By using this theory, the gas pressure for the experimental velocity was determined.

**Figure 13:**
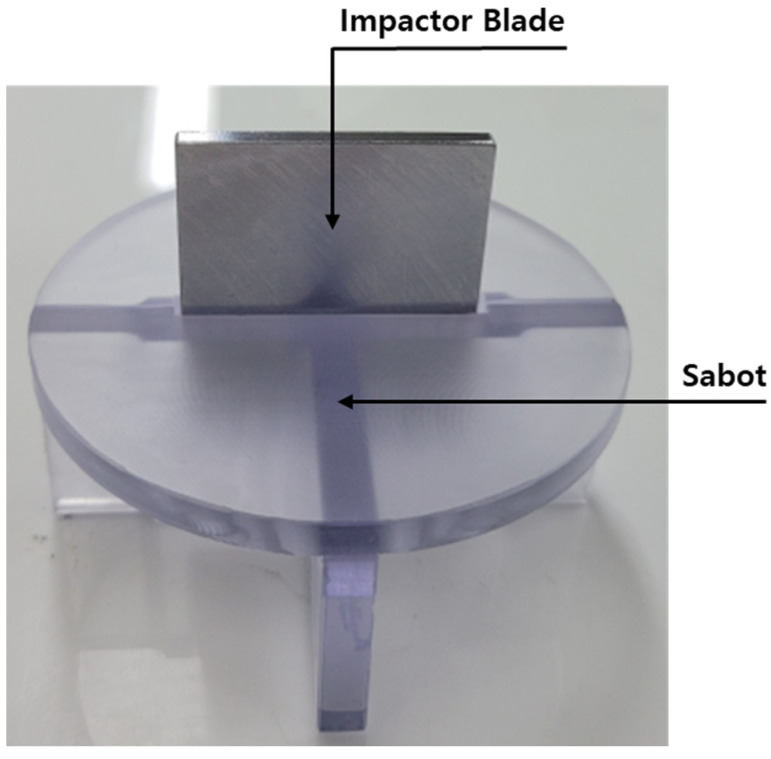
Sabot with impactor blade.

**Figure 14:**
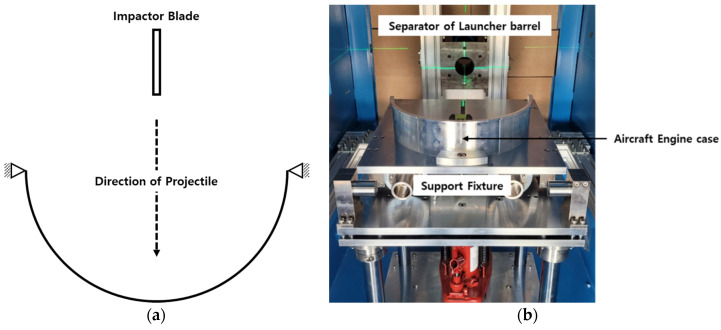
Projectile impact angle schematic for ballistic impact experiments: (**a**) schematic of experiment; (**b**) target engine case assembly.

The ballistic impact experiment involved a 0.4 m diameter specimen made of aluminum 6061-T6. The projectile used was a steel blade with a mass of 0.055 kg measuring 0.05 m by 0.04 m. Helium was employed as the pressurizing gas. To prevent projectile shock, the experiments were conducted in a vacuum environment at −0.95 bar. Additionally, a sabot (as shown in Figure 13) was designed to maintain the projectile’s trajectory. The impact angle of the projectile was carefully controlled (as depicted in Figure 14). The experimental results were used to assess the damage profile and the corresponding projectile velocity post-impact. We measured the projectile’s velocity in both nonpenetration and penetration scenarios on a cylindrical shell with a 5 mm thickness, and the damage pattern was confirmed after the experiment.

## 4. Results

**Figure 15:**
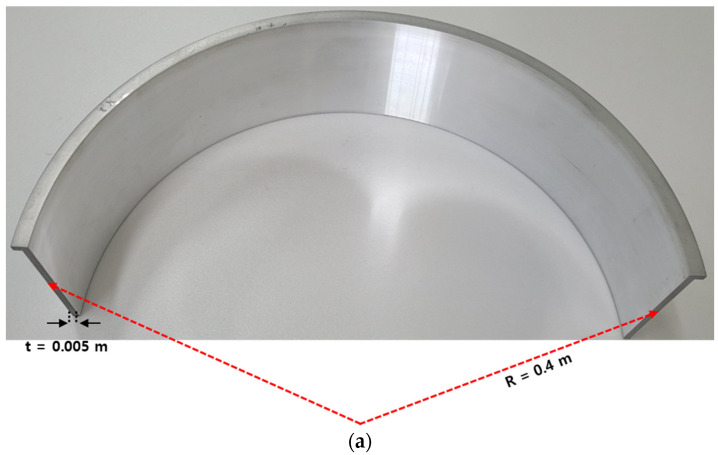

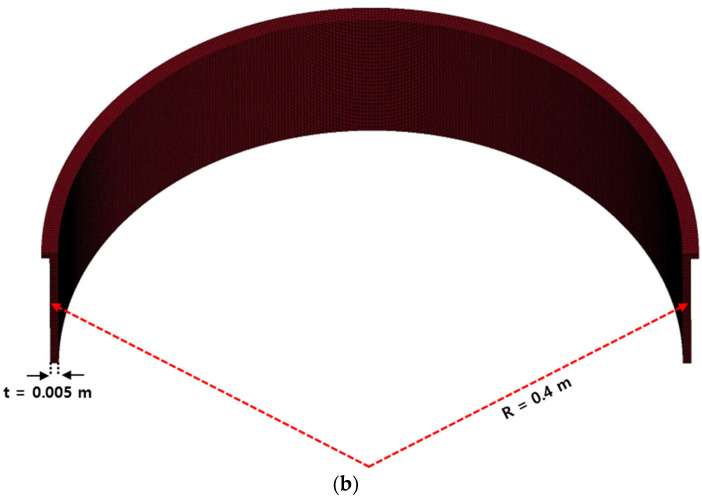
Blade containment case model for ballistic impact analysis: (**a**) aluminum 6061 for aircraft engine case; (**b**) analysis model of the aluminum 6061 for aircraft engine case.

Ballistic impact analysis, which is used to validate the coefficients of the MJC material model and the CL fracture model, was conducted using the LS-DYNA, R12.0.0 program. Figure 15 illustrates aluminum 6061-T6 used for the cylindrical shell. The element erosion condition (CONTACT ERODING SINGLE SURFACE) was applied to a solid element for fracture shape simulation. The number of elements in the analysis model was 165,000. The impact characteristics according to the shape of the target were analyzed by comparing the analysis and experimental results. A projectile velocity was 105–244 m/s. The penetration process of the blade was confirmed through numerical analysis, which showed that bending occurred on the surface of the target because of the impact load of the projectile when the projectile hit the case for the first time. Element damage continues to occur over time in the impact zone of the target surface. Accordingly, cracking occurs on the surface of the target when the elements that exceed the fracture strain fall off. 

**Figure 16:**
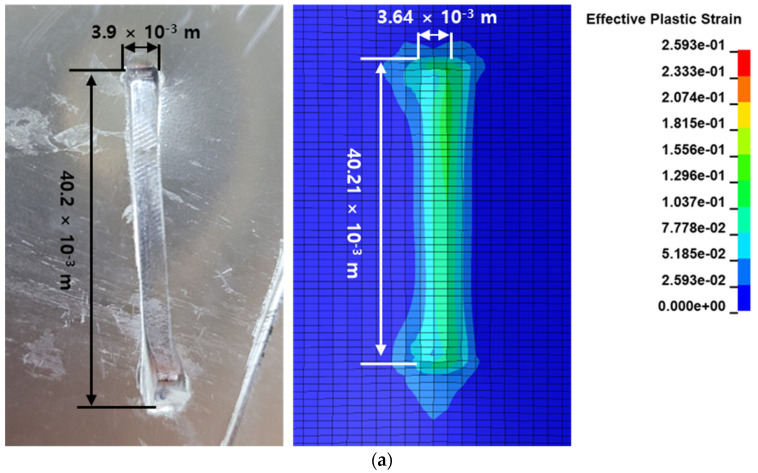

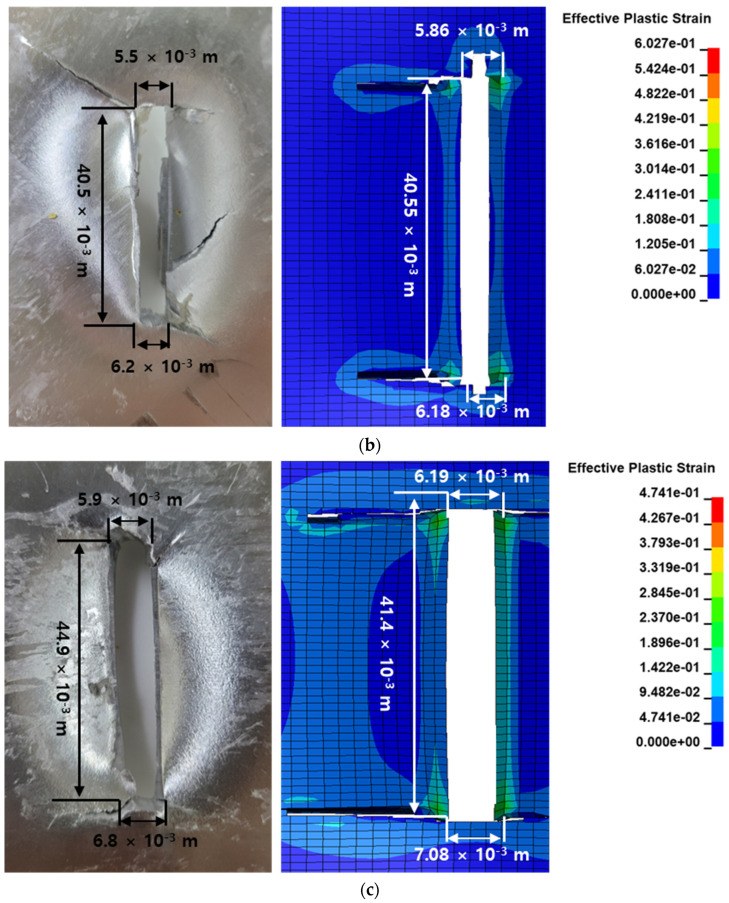
Comparisons of the damage patterns for engine case: (**a**) ballistic impact velocity: 105.4 m/s; (**b**) ballistic impact velocity: 163.8 m/s; (**c**) ballistic impact velocity: 244.3 m/s.

Figure 16 illustrates the resultant damage shapes obtained from the analysis and experimental data, confirming a maximum error of approximately 6% in the damage shape. The shape closely matched the impact velocity during ballistic tests. The shape resulting from ballistic impacts at various velocities was quantified. This shape, observed under the high-speed metallic material impact, was corroborated by both analysis and experimentation. The assessment of this outcome was based on the penetration outcomes from high-velocity ballistic impacts, as outlined in Table 5. The target’s failure results encompassed both complete and incomplete penetration. While the analysis initially predicted excessive damage, it was subsequently verified to be relatively accurate in determining penetration at specific impact velocities. The combined analysis and experimental results provided validation for the methods employed in analyzing the target’s ballistic impact behavior with respect to its shape.

## 5. Conclusions

This study introduces a novel approach to determining the strain rate coefficient in ballistic impact analysis. Traditional dynamic experiments are typically conducted using the SHPB technique, but it is limited to a strain rate of 10^4^ s^−1^. To address this limitation, we propose a method based on ball impact energy calculation to obtain a more accurate strain rate coefficient. Moreover, our method allows for the determination of the coefficient at a significantly higher strain rate of 12,000 s^−1^ compared to the SHPB experiments.

The coefficients for the modified Johnson–Cook (MJC) and Cockcroft–Lathan (CL) fracture models are derived from both quasi-static experiments using an MTS-810 and dynamic experiments using our proposed method. Our ballistic impact equipment utilizes a gas gun mechanism to propel projectiles, which is validated through penetration experiments, revealing errors of 3.9%, 3.7%, and 0.7% based on plate thickness.

Furthermore, we manufactured a cylindrical shell measuring 5 mm in thickness, constructed from aluminum 6061-T6. We proceeded to predict the damage pattern across a range of impact velocities. Subsequently, these coefficients are utilized in simulations employing LS-DYNA, R12.0.0. The validation of blade containment is achieved by comparing the anticipated damage pattern with the outcomes of experimental tests.

This research holds the potential to become an invaluable technique for assessing the structural integrity of aircraft engine cases in forthcoming investigations. Nonetheless, it is important to highlight that the current methodology does not consider the influence of temperature. Given the possibility of localized high temperatures in the impact area, accounting for temperature effects could significantly enhance the accuracy of material behavior predictions during high-velocity impacts. Consequently, in future research, we intend to delve deeper into the impact of temperature on the behavior of aluminum 6061-T6 in high-velocity scenarios.

## Figures and Tables

**Table 1:** Results of the ball impact experiment.

No.	Velocity (m/s)	Velocity (m/s)
1	4.166	71.42
2	4.985	75.39
3	4.743	69.62
Average	4.743	72.14

**Table 2:** The coefficients of the modified Johnson–Cook and Cockcroft–Lathan fracture models.

Initial Yield Stress, *A* (MPa)	245	Strain Hardening Parameter, *B* (MPa)	233
Strain Hardening Exponent, *n*	0.34	Strain Rate Sensitivity Parameter, *C*	0.03
Reference Plastic Strain Rate, s^−1^	0.01	Strain Energy, Wc (MPa)	58.5

**Table 3:** Material properties of aluminum 6061.

Material Properties (Aluminum 6061)			
Density (kg/m^3^)	2.713	Poisson’s Ratio	0.33
Modulus of Elasticity (GPa)	68.3	Specific Heat Capacity (J/(kg·K))	890
Shear Modulus (GPa)	27.7		

**Table 4:** Comparison of ballistic impact results for the verification of coefficients.

No.	Thickness (mm)	Experiment (m/s)	Analysis (m/s)	Error (%)
1	3	103.9 (Penetration)	92 (Critical Velocity)	3.9
		87.5 (Nonpenetration)		
2	6	166.7 (Penetration)	154 (Critical Velocity)	3.7
		153.2 (Nonpenetration)		
3	8	211.5 (Penetration)	200 (Critical Velocity)	0.7
		191.3 (Nonpenetration)		

**Table 5:** Ballistic impact experimental results of the aircraft engine case.

No.	Velocity (m/s)	Containment Evaluation Result
1	244.3	Un-Containment: Complete Penetration
2	163.8	Un-Containment: Non-Complete Penetration
3	105.4	Containment: Rebound, Nonpenetration

## Data Availability

The datasets used and/or analysed during the current study available from the corresponding author on reasonable request.
